# The Nutraceutical Genistein-Lycopene Combination Improves Bone Damage Induced by Glucocorticoids by Stimulating the Osteoblast Formation Process

**DOI:** 10.3390/nu14204296

**Published:** 2022-10-14

**Authors:** Federica Mannino, Tommaso D’Angelo, Giovanni Pallio, Antonio Ieni, Igor Pirrotta, Domenico Antonio Giorgi, Alessandro Scarfone, Silvio Mazziotti, Christian Booz, Alessandra Bitto, Francesco Squadrito, Natasha Irrera

**Affiliations:** 1 Department of Clinical and Experimental Medicine, University of Messina, Via C. Valeria Gazzi, 98125 Messina, Italy; 2 Department of Biomedical, Dental, Morphological and Functional Imaging Sciences, University of Messina, Via C. Valeria Gazzi, 98125 Messina, Italy; 3 Department of Radiology and Nuclear Medicine, Erasmus MC, Dr. Molewaterplein 40, 3015 GD Rotterdam, The Netherlands; 4 Department of Human Pathology in Adult and Developmental Age “Gaetano Barresi”, University of Messina, Via C. Valeria Gazzi, 98125 Messina, Italy; 5 Division of Experimental Imaging, Department of Diagnostic and Interventional Radiology, University Hospital Frankfurt, Theodor-Stern-Kai 7, 60590 Frankfurt am Main, Germany

**Keywords:** osteoporosis, GIO, genistein, lycopene, nutraceuticals

## Abstract

Chronic glucocorticoid (GC) therapy is the most common cause of iatrogenic osteoporosis and represents an important risk factor for osteoporosis and bone fractures. New therapeutic approaches are required in order to treat osteoporosis and reduce the side effects related to the use of anti-osteoporotic drugs. In this context, previous studies reported the efficacy of some isoflavones and carotenoids, such as lycopene and genistein, on the reduction of the risk of fracture related to osteoporosis. The aim of this study was to investigate the effects of a combined oral treatment, consisting of genistein and lycopene, in an experimental model of glucocorticoid-induced osteoporosis (GIO). GIO was induced by subcutaneous injection of methylprednisolone (MP, 30 mg/kg) for 60 days, whereas the control group (Sham) received saline solution only. Following induction, MP animals randomly were assigned to receive alendronate, genistein, lycopene, or the association of genistein and lycopene or saline solution for additional 60 days together with MP. Femurs obtained from the Sham group were used for osteoblasts extraction; they were then incubated with dexamethasone (DEX) for 24 h to be then treated with lycopene or genistein or the association of lycopene and genistein for an additional 24 h. Treatments with lycopene and genistein restored the impaired mineralization of cells observed following DEX treatment and stimulated osteoblast differentiation by increasing the depressed expression of bALP and RUNX2 (*p* < 0.0001). Wnt5a, β-catenin, and Nrf-2 expression were significantly increased following genistein and lycopene treatment (*p* < 0.0001), thus confirming their antioxidant activity as well as their ability in stimulating osteoblast function, mostly when genistein and lycopene were used in association. The combined treatment of genistein and lycopene improved the bone damage induced by glucocorticoids and significantly restored the normal architecture of bones as well as adequate interconnectivity of bone trabeculae, thus increasing bone mineral density parameters. The obtained data demonstrated that genistein and lycopene but in particular their association might prevent GC’s adverse effects, thus stimulating bone formation and reducing bone resorption, improving bone structure and microarchitecture, through different molecular pathways, such as the Wnt/β-catenin and the Nrf-2 signaling.

## 1. Introduction

The dynamic balance of bone metabolism is a physiologic process throughout the lives of humans [1]; the sophisticated equilibrium of bone formation and resorption is maintained by osteoblasts and osteoclasts which are the main cells involved in the release of matrix components and proteinases, respectively [2]. The alteration of this balance may be physiologically observed in aged patients but may also be responsible for the appearance of metabolic bone diseases, including osteoporosis [3]. Osteoporosis is a growing health problem worldwide that affects patients of all races and genders, although Caucasians, women, and older people are the most affected [4]. Menopause, inadequate diet together with other incorrect lifestyle habits such as sedentarism, smoking, and alcohol consumption may represent contributing causes for osteoporosis [5]. Among the possible causes, Glucocorticoid (GCs) administration may also result in osteoporosis induction, thus increasing the risk of fractures [6]. Although GCs are generally used for the treatment of immune-mediated and allergic diseases, their use may be strongly related to the adverse effects on bone by reducing bone mineral density (BMD); increased fracture risk was observed on the hips and spines of patients following corticosteroid administration [5]. Glucocorticoid-induced osteoporosis (GIO) pathogenesis is complex and involves different molecular pathways such as the NF-κB ligand (RANKL)/osteoprotegerin (OPG) signaling, whose activation stimulates osteoclastogenesis and inhibits osteoblast differentiation, thus reducing matrix production and increasing mechanisms of cell death in osteoblasts and osteocytes [7]. For this reason, RANKL represents a pharmacological target, and denosumab is prescribed as a RANKL inhibitor for the management of osteoporosis, despite its use as well as that of other anti-osteoporotic drugs, such as bisphosphonates and calcitonin, is related to adverse effects and regular infusion appointments are required following long-term administration which may represent a discomfort for patients [8]. In this context, the scientific challenge is to find new therapeutic approaches adequate for long-term use with negligible side effects. Previous studies already reported the efficacy of isoflavones and carotenoids on bone health: a high dietary intake of both isoflavones and/or carotenoids may improve BMD, thus reducing the osteoporotic fracture risk by balancing different cell signaling [9,10]. Among isoflavones, genistein, which is a selective estrogen receptor modulator (SERM) and binds the estrogen receptor beta (ERβ), was already used in postmenopausal women and its administration was found to be not only effective in increasing BMD but also safe [11]. Moreover, the beneficial effects of lycopene, due to its antioxidant activity and singlet oxygen separation capacity, have been demonstrated by in vitro, in vivo, and clinical studies [12]. Furthermore, lycopene intake was effective in reducing bone loss and bone resorption markers in postmenopausal women [13].

Therefore, on the basis of these scientific assumptions and clinical evidence, the aim of the present study was to comprehensively investigate the effects of a combined oral treatment, consisting of genistein and lycopene, in both in vitro and in vivo models of glucocorticoid-induced osteoporosis.

## 2. Materials and Methods

### 2.1. Experimental In Vivo Model of Glucocorticoid-Induced Osteoporosis

Female Sprague–Dawley rats (*n* = 60; Charles River, Calco, Italy) 5 months of age and weighing between 250–275 g were used in this study. Upon arrival, animals were housed (3–4 rats per cage) in the Animal Facility of the Department of Clinical and Experimental Medicine of the University of Messina and provided with a standard diet ad libitum as well as free access to water. Rats were monitored daily and maintained in a 12 h light/darkness cycle and a temperature of 24 °C. The present study was approved by the Ethics Committee of Messina University and by the National Ethics Committee for Research Animal Welfare of the Italian Ministry of Health (authorization no. 679/2019-PR on 7 October 2019). All the experimental procedures complied with the ARRIVE guidelines [14] and were compliant with the Italian Guidelines for Care and Use of Laboratory Animals (D.L.116/92) and with the European Directive (2010/63/EU).

The in vivo model of glucocorticoid-induced osteoporosis (GIO) was induced by subcutaneous (s.c.) injection of methylprednisolone (MP; Sigma–Aldrich, Milan, Italy) for 60 days at the dose of 30 mg/kg which represents the human equivalent dose (7.5 mg). Following induction, MP animals (*n* = 50) were randomly assigned to receive Alendronate 1 mg/kg (MP + Ale; *n* = 10), genistein 5 mg/kg (MP + GEN; *n* = 10), lycopene 10 mg/kg (MP + LYCO; *n* = 10), or the association of genistein and lycopene (which were sequentially administered) (MP + GEN + LYCO; *n* = 10) or saline solution (MP; *n* = 10). All treatments were daily administered per os through gavage and lasted additional 60 days simultaneously with MP (s.c.). The Sham group (SHAM; *n* = 10) received saline throughout the experimental protocol (120 days). At the end of the experimental procedure, all animals were sacrificed by cervical dislocation, and femurs were collected for the analyses described below. 

### 2.2. Primary Osteoblast Culture and Extraction

Femurs obtained from the Sham group were used for primary osteoblasts extraction. Once collected, epiphyses of femurs were cut and put aside in order to remove bone marrow inside the diaphysis by flushing with PBS. Little pieces of bone were then obtained by cutting diaphysis and after several washes with PBS, they were incubated with collagenase II (2 mg/mL) diluted in culture medium (DMEM) at 37 °C for 2 h on a shaker to promote all soft tissue removal. After additional washes with culture medium (DMEM plus 10% FBS, 100 U/mL penicillin, 50 μg/mL streptomycin sulfate; Sigma–Aldrich, Milan, Italy) bone pieces were cultured and incubated in Petri dishes at 37 °C of temperature and 5% of CO_2_. The culture medium was changed whenever needed and cells started to migrate from bone pieces following 2–3 days of incubation; bones were removed on day 7 and cells were collected on day 15 for further experiments.

### 2.3. In Vitro Model of Glucocorticoid-Induced Osteoporosis

The obtained primary osteoblasts were incubated with 1 μM of dexamethasone (DEX, Sigma–Aldrich, Milan, Italy) for 24 h to induce an in vitro model of osteoporosis, as previously described [15]. After incubation, cells were treated with lycopene at the concentration of 0.5 μM or genistein at the concentration of 25 μM or with the association of lycopene and genistein for an additional 24 h. Cells were collected for Alizarin Red S (ARS) staining and Reverse transcription-quantitative (RTqPCR) analysis.

### 2.4. Alizarin Red S Staining

Once collected, osteoblasts were fixed with paraformaldehyde (4%) for 30 min at 37 °C in the dark and then stained with ARS at the concentration of 1% (Sigma–Aldrich, Milan, Italy) for 5 min at room temperature. The formed calcification nodules were detected under an inverted light microscope and images were captured at ×10 magnification.

### 2.5. RT-qPCR 

cDNA was reverse transcribed (High-Capacity cDNA Archive Kit; Thermo Fisher Scientific, Waltham, MA, USA) from 2 μg RNA extracted from cells using TRIzol LS reagent (Invitrogen, Carlsbad, CA, USA) according to the manufacturer’s protocols. 

Real-time PCR analysis was used to study the mRNA expression obtained from the different experimental groups, as previously described, by using specific primers reported in Table 1. 20 µL reactions containing 1xSYBR^®^ Select Master Mix (Thermo Fisher Scientific, Waltham, Massachusetts, USA), primers (10 µM) and cDNA were run in duplicate in 96-well plates using QuantStudio 6 Flex Real-Time PCR System (Applied Biosystems, Foster City, CA, USA). Data were reported using the 2^−ΔΔCT^ relative quantification method and GAPDH was used as a control. Graphs represent the values as fold changes relative to the control cells [16,17]. 

### 2.6. Histology

Bone tissue was collected by leg disarticulation at the hip, knee, and ankle. Once femurs were removed, they were immediately cleaned to remove soft tissues and fixed in 10% neutral-buffered formalin. A decalcifying solution composed of 8% HCl from 37% (*v*/*v)* concentrate and 10% formic acid from 89% (*v*/*v*) concentrate dissolved in PBS was used for ∼24 h at 37 °C and later samples were dehydrated in graded ethanol to be embedded in paraffin. Hematoxylin–eosin staining was performed on 5-μm thick bone sections and observed under light microscopy. 

### 2.7. Microcomputed Tomography (µCT) Measurements

The effects of treatments on bone microstructure were evaluated using µCT (SkyScan1174; Bruker; Kontich, Belgium). The femoral bones were extracted for ex-vivo µCT at the end of the experimental procedure (120 days) and stored at −20 °C until scanning. 

The excised femurs were defrosted at room temperature and then scanned parallel to the sagittal and coronal plane.

Scanning parameters were set as follows: camera pixel size (15.45 µm); tube voltage (50 kV), tube current (800 µA); image pixel size (11.1 µm); exposure (500 ms); rotation step (0.8°); 180° tomographic rotation with random movement; filter (Al 0.5 mm). 

Image reconstruction was performed using NRecon software (Bruker; Kontich, Belgium), through Hamming-filtered back projection, with the following parameters: smoothing (4); smoothing kernel (2—Gaussian); ring artifact correction (4); beam hardening correction (41%); grey thresholds (82–255). 

µCT images were resliced parallel to the axial plane of the femoral neck, and the cortical and trabecular bone microarchitecture analysis was performed on 350 consecutive slices.

CTAn Skyscan 1275 software (Bruker; Kontich, Belgium) was preliminarily used to separate the cortical and trabecular volumes. To analyze the trabecular bone microarchitecture the bone volume fraction (bone volume/total volume, tBV/TV, unit = %), trabecular thickness (Tb.Th, unit = µm), trabecular separation (Tb.Sp, unit = µm) were measured by means of Batman tool. Structure model index (SMI), which assesses the change in surface curvature occurring when a structure varies from spherical (SMI  =  4) to cylindrical (SMI  =  3) to planar (SMI  =  0), was also considered to measure rods and plates morphology in trabecular bone.

Bone mineral density (BMD, unit = mg/cm^3^) was determined from the trabecular volume, using hydroxyapatite phantom rods of 4 mm in diameter with known BMD (0.25 gHA/cm^3^ and 0.75 gHA/cm^3^) as reference. Subsequently, a separate analysis and quantification of the cortical bone microstructure were performed by evaluation of cortical bone fraction (cBV/TV, unit = %) [18,19,20].

Results of bone microarchitecture parameters from the right and left sides were averaged to determine differences amongst groups.

### 2.8. Statistical Evaluation

All data are expressed as means ± Standard Deviation (SD). Both for in vitro and in vivo experiments the differences between the groups were evaluated and analyzed using one-way ANOVA with the Tukey post-test. A *p* value of less than 0.05 was considered significant. SPSS Statistics for Windows v22.0 was used for statistical analysis (SPSS, Inc., Chicago, IL, USA) and GraphPad Prism was used to create the graphs (Version 8.0 for macOS, San Diego, CA, USA).

## 3. Results

### 3.1. Genistein and Lycopene Treatment Stimulates Osteoblast Activity 

**Figure 1:**
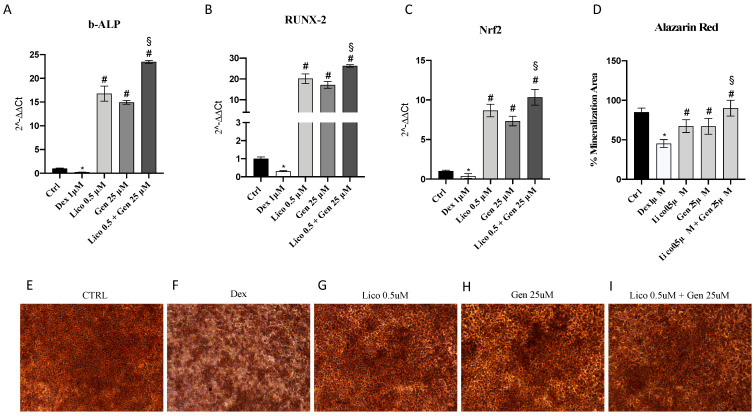
The graphs (**A**–**C**) represent b-ALP, RUNX-2, and NRF2 mRNA expression evaluated by q-PCR in primary osteoblasts treated with lycopene or genistein or lycopene and genistein following dexamethasone challenge for 24 h. The figures (**E**–**I**) show Alizarin Red S staining; primary osteoblasts incubated with dexamethasone for 24 h exhibit a reduction of mineralized matrix formation, demonstrated by the red color attenuation (**F**); cells treated with lycopene, genistein, and their association are characterized by a strong alizarin red accumulation, resulting from an increased mineralized matrix formation (**G**–**I**). Panel (**D**) shows the % of Mineralization Area evaluated in primary osteoblasts through Alizarin Red staining. The data are expressed as the means and SD. * *p* < 0.0001 vs. Ctrl; # *p* < 0.0001 vs. Dex; § *p* < 0.0001 vs lycopene and genistein.

Bone ALP and Runx2 expression was investigated as hallmarks of osteoblast differentiation. Dexamethasone treatment reduced both bALP and Runx2 expression compared to control cells (not treated with DEX). Both treatments with genistein or lycopene significantly increased bALP and Runx2, however, an even more significant increase was observed following osteoblasts incubation with the association of genistein and lycopene (Figure 1A,B). 

Alizarin Red S staining confirmed that DEX weakened mineralized matrix formation in osteoblasts, as observed by the red color attenuation (Figure 1F). Treatments with genistein or lycopene and especially the association of genistein and lycopene restored mineralization levels in treated cells (Figure 1G–I).

The antioxidant Nrf-2 transcription factor was significantly increased following genistein and lycopene treatment but the adjunction of the combination of genistein and lycopene further augmented the hampered reduction of Nrf-2 in DEX-challenged osteoblasts (Figure 1C).

**Figure 2:**
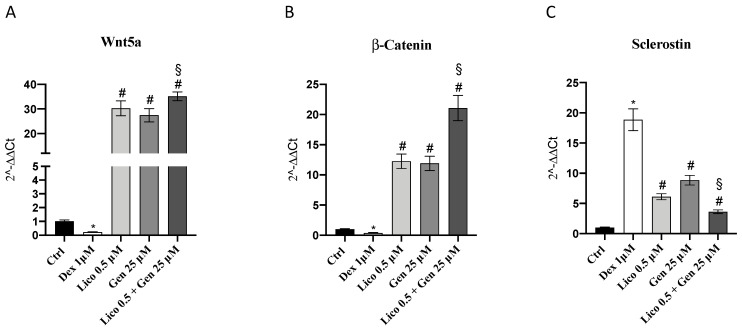
The graphs (**A**–**C**) represent Wnt5a, β-Catenin, and Sclerostin mRNA expression evaluated by q-PCR in primary osteoblasts treated with lycopene or genistein or lycopene and genistein following dexamethasone challenge for 24 h. The data are expressed as means and SD. * *p* < 0.0001 vs. Ctrl; # *p* < 0.0001 vs. Dex; § *p* < 0.0001 vs lycopene and genistein.

Osteoblast activity is also regulated by other molecular pathways, such as the Wnt/β-catenin signaling. Additionally, Wnt5a and β-catenin expression were significantly increased following genistein and lycopene treatment; the concomitant administration of genistein and lycopene significantly increased the expression of both targets compared to the treatments alone. Dexamethasone increased sclerostin expression, which is an inhibitor signal for osteoblast activity, whereas the treatments alone but in particular the association of genistein and lycopene significantly decreased sclerostin levels compared to DEX-treated cells. These results suggested that both genistein or lycopene treatment and even more the association of genistein and lycopene may stimulate osteoblast activity in depressed conditions like that reproduced in the present in vitro model of osteoporosis induced by dexamethasone administration (Figure 2). 

### 3.2. The Association of Genistein and Lycopene Improves Bone Microarchitecture and Volumetric BMD

**Figure 3:**
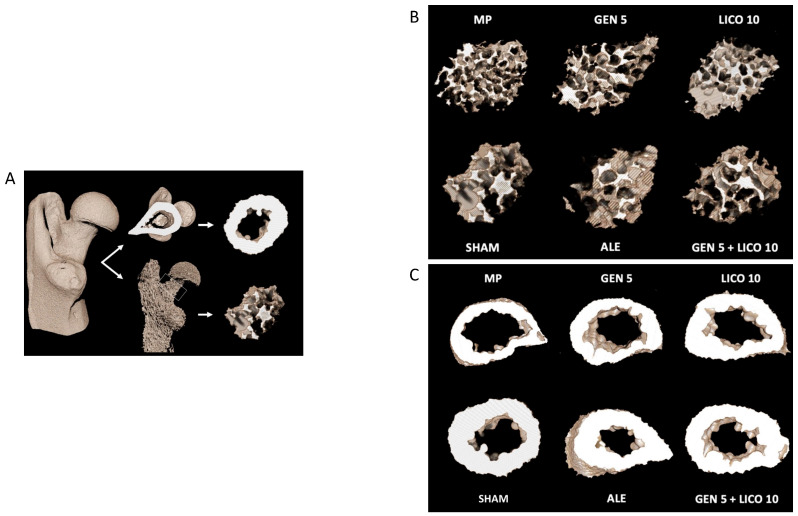
µCT three-dimensional reconstruction image of the proximal epiphysis of a rat femur. The scheme shows how images were post-processed in order to separate trabecular and cortical volumes, prior to proceeding with the femoral neck morphometric analysis and BMD assessment (**A**). µCT three-dimensional images of the trabecular (**B**) and cortical (**C**) bone microarchitecture of the femoral neck. (**A**) Trabecular thickness is increased in the ALE and GEN LICO group, compared to the MP vehicle. Similarly, there is a rods-to-plates transition in the same groups, compared to the vehicle. (**B**) Cortical thickness and bone fraction are consensually increased in ALE and GEN LICO groups compared to MP vehicles.

The analysis of 3D µCT parameters of bone structure at femoral proximal epiphysis showed significant differences in bone microarchitecture among the different experimental groups (Figure 3).

A significant bone deterioration was detected in osteoporotic rats (MP group) compared to controls (Sham), which showed looser trabecular bone microarchitecture, reduced cortical bone fraction, and endosteal cortical erosion (Figure 3). 

**Figure 4:**
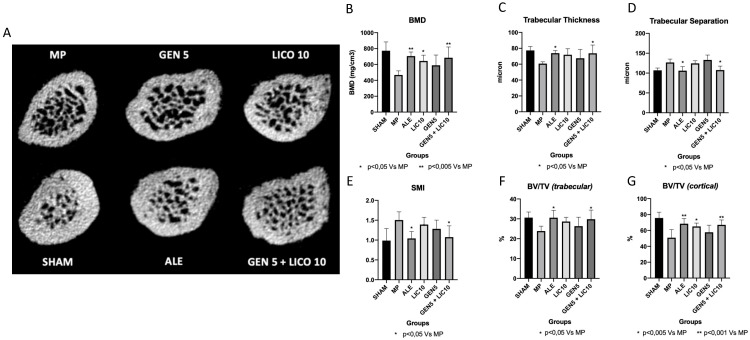
µCT axial images obtained at the level of the femoral neck (**A**); bone mineral density (**B**); trabecular thickness (**C**); trabecular separation (**D**); structure model index (**E**); trabecular bone volume/total volume (**F**); cortical bone volume/total volume (**G**). Images show reduced cortical bone thickness with areas of endosteal cortical erosion in the MP vehicle, with an increase in trabecular space and a reduction in trabecular thickness. On the contrary, ALE, and GEN LICO groups showed an increased cortical and trabecular thickness, with a reduction of the trabecular spaces.

Femoral neck analysis (Figure 3) showed that mineralization increased following oral administration of alendronate, genistein, lycopene, and combined treatment of genistein and lycopene compared to MP animals. A significant increase of BMD (Figure 4B) was observed in the alendronate group and in animals treated with the association of genistein and lycopene, where a 5% of increment of the trabecular bone fraction and 15% increment of the cortical bone fraction with increased Tb.Th parameters were observed (Figure 4C). Conversely, Tb.Sp and SMI, both inversely correlated to the mineralization quality of the trabecular bone, showed a significant decrease in these groups compared to the MP vehicle (Figure 4D,E). 

All 3D morphometric parameters did not show any significant difference when nutraceuticals were used alone compared to the MP group, either for the trabecular and cortical bone compartments, whereas a significant improvement was observed following the administration of the association of genistein and lycopene.

### 3.3. The Combined Treatment of Genistein and Lycopene Improves Bone Structure

**Figure 5:**
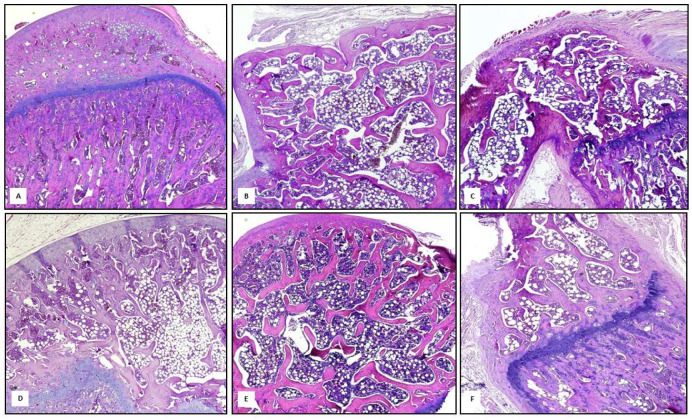
The images show Hematoxylin–eosin staining (original magnification 10×) performed on bone sections from Sham group (**A**), MP group (**B**), MP + Ale group (**C**), MP + GEN group (**D**), MP + LYCO group (**E**) and MP + GEN + LYCO group (**F**).

Bone tissue samples obtained from the Sham group showed a normal compact bone structure of diaphysis composed of parallel columns of Haversian system with concentric rings of calcified matrix and spongy component, thus exhibiting a homogeneous trabecular network with regular lamellar thick (Figure 5A). In contrast, the osteoporotic bone of MP animals was characterized by cortical and trabecular bone loss, with thin and disconnected unsupported vertical trabeculae and surrounding fatty bone marrow spaces (Figure 5B). Animals treated with alendronate showed a marked decrease in the thickness of the compact bone, loss of interconnectivity and thinning of the trabeculae showing widened intertrabecular spaces compared with the sham group (Figure 5C). Similar features were observed in genistein-treated rats, however, the treatment with genistein slightly increased thin disconnected trabeculae even if intertrabecular space enlargement may be still detected (Figure 5D). Lycopene treatment significantly increased bone trabecular thickness, with smaller resorption spaces arranged in anastomosing bone lamellae (Figure 5E). Finally, the combined treatment of genistein and lycopene significantly restored normal architecture and adequate interconnectivity between the bone trabeculae, narrowing of bone marrow spaces and thickening of trabecular bone may be seen (Figure 5F).

## 4. Discussion

Glucocorticoid-induced osteoporosis is a well-known cause of iatrogenic osteoporosis, in particular following long-term GC use. Glucocorticoids may influence osteoblasts viability and differentiation, thus inducing osteoclastogenesis and producing a significant imbalance of antiresorptive molecules, such as OPG and RANKL [21]. To switch off bone mass loss resulting from osteoporosis, the stimulation of osteoblasts/osteocytes differentiation, function, and survival as well as the suppression of osteoclasts activity may represent an effective strategy, particularly during chronic GC treatment and menopause. RUNX2 transcription factor is a key regulator of osteoblast formation and function thanks to its role in stimulating bone formation genes [22]. In this context, different molecular pathways and transcription factors are involved in RUNX2 activation in osteoblasts, such as the Nrf2 transcription factor and the Wnt/β-catenin signaling [23]. Once the canonical Wnts bind their specific receptors and co-receptors, β-catenin interacts with the LDF1/TCF1 transcription factors and the activated Smads in order to promote RUNX2 gene transcription [24]. Oxidative stress may significantly reduce osteoblasts differentiation and proliferation in favor of osteoclast activity thus promoting bone resorption [25]; for this reason, the use of antioxidants which might act as ROS scavengers and might induce antioxidant factors, such as Nrf-2, might be curative for osteoporotic damage. In this context, previous studies indicated that carotenoids and isoflavones were effective in maintaining bone mineral status and although their mechanism of action is not fully understood, many of them may have a significant antioxidant potential [26,27]. Different in vitro and in vivo clinical studies reported the beneficial effects of the carotenoid lycopene and the isoflavone genistein on bone health and supported the scientific approach of the present study based on the use of an association of genistein and lycopene in the well-known experimental models of glucocorticoid- and dexamethasone-induced osteoporosis. Both genistein and lycopene significantly increased the depressed expression of bALP and RUNX2 observed in DEX- stimulated cells and this positive effect was most evident when genistein and lycopene were used together. Additionally, Wnt5a, β-catenin, and Nrf-2 expression were significantly increased both with genistein and lycopene treatment alone and with their concomitant administration, thus confirming their antioxidant activity as well as their ability in stimulating osteoblast function through Nrf-2 and Wnt/β-catenin signaling modulation mostly when genistein and lycopene were used in association. Sclerostin produced by osteocytes antagonizes Wnt signaling and reduces bone formation through inhibition of the terminal differentiation of osteoblasts, thus inducing their apoptosis [28]. Dexamethasone challenge increased sclerostin expression in primary osteoblasts whereas the treatments alone, but in particular genistein and lycopene in association significantly decreased sclerostin levels compared to DEX-challenged cells and confirmed the ability of these two nutraceuticals in promoting osteoblast function and quality. In addition, Alizarin Red S staining confirmed that genistein or lycopene, and especially the combined use of genistein and lycopene restored the weakened mineralization levels observed in the DEX group. To confirm GC-induced bone loss, the effects of methylprednisolone administration were evaluated in rats, and genistein or lycopene were administered with two low doses (5 and 10 mg/kg, respectively), chosen in accordance with previous experiments [29,30]. 

Since the positive effects of genistein and lycopene were already demonstrated in osteoporosis experimental models as well as in clinical trials [11,31], the novelty of the present experiment is based on the use of sequential administration of genistein and lycopene at two low doses in order to evaluate their greater efficacy in association than their use alone. Significant differences were observed in bone structure and microarchitecture between the experimental groups and in particular, bone loss was detected at cortical and trabecular levels in the MP group as well as an increased number of trabeculae, thus confirming MP-induced osteoporosis. In fact, MP animals showed a significant reduction in BMD levels compared to Sham rats. The treatment with alendronate, used as a gold standard [32], and with genistein or lycopene increased mineralization and restored bone structure, in terms of bone loss and separated trabeculae reduction. However, genistein alone slightly increased thin disconnected trabeculae whereas lycopene was able to increase bone trabecular thickness. These data are in accordance with previous studies that showed genistein and lycopene efficacy on bone microarchitecture although different experimental models and doses were used [33,34]. Surprisingly, the combined treatment of genistein and lycopene significantly restored normal architecture and adequate interconnectivity between the bone trabeculae, thus increasing BMD levels. 

The data described so far confirm that glucocorticoids may affect bone metabolism, thus inducing bone resorption and suppressing bone formation, as demonstrated by µCT and histological analyses. Genistein and lycopene but in particular the association of both might prevent GCs’ adverse effects, thus stimulating bone formation and reducing bone resorption, improving bone structure and microarchitecture. These results are supported by in vitro experiments that showed that both nutraceuticals were able to stimulate osteoblasts activity through different molecular pathways, such as the Wnt/β-catenin and the Nrf-2 signaling. Recently the attention of researchers has focused on the clinical development of osteoanabolic agents for the management of osteoporosis; the final goal is to ameliorate bone strength by positively modulating bone modeling and remodeling. Indeed, the rational therapeutic strategy aims at normalizing bone strength by replacing bone mass loss and by improving the disturbed skeletal architecture. Bisphosphonates and denosumab are the most used anti-resorptive drugs which reduce both bone resorption and partial bone formation, thus causing a positive bone balance and ameliorating, in turn, bone mineral density, and bone strength parameters. The weakness of this therapeutic strategy stems from the lack of effects on the bone formation that does not allow a restoration of the altered microarchitecture of the trabecular or cortical bone. Only the osteoanabolic and bone-forming drugs teriparatide (the 1–24 PTH fragment) and romosozumab (an antibody against sclerostin) may accomplish this task, thus they are currently subjected to intense clinical investigation in patients with osteoporosis, especially those with a high risk of fracture. We are tempted to speculate that the genistein-lycopene combination falls in the category of osteoanabolic agents and may represent a natural and safe alternative therapy to both teriparatide and romosozumab. However, this hypothesis needs to be deeply explored and confirmed in randomized clinical trials.

## Figures and Tables

**Table 1:** Primer list.

Primer	Forward	Reverse
b-ALP	GCTGATCATTCCCACGTTTT	CTGGGCCTGGTAGTTGTTGT
RUNX-2	CCCAGCCACCTTTACCTACA	TATGGAGTGCTGCTGGTCTG
Wnt5a	CAAATAGGCAGCCGAGAGAC	CTCTAGCGTCCACGAACTCC
β-Catenin	GTGCAATTCCTGAGCTGACA	CTTAAAGATGGCCAGCAAGC
Sclerostin	GCCGGACCTATACAGGACAA	CACGTAGCCCAACATCACAC
Nrf2	CTCGCTGGAAAAAGAAGTGG	CCGTCCAGGAGTTCAGAGAG

## Data Availability

The data presented in this study are available on request from the corresponding author.
